# Age related differences in balance approached by a novel dual-task test of anticipatory postural control strategies

**DOI:** 10.1371/journal.pone.0218371

**Published:** 2019-06-27

**Authors:** Uffe Laessoe, Camilla Bille Larsen, Line Noerkjaer Schunck, Line Jensen Lehmann, Halla Iversen

**Affiliations:** 1 Physical Therapy Department, University College of Northern Denmark, Aalborg, Denmark; 2 Research and Development Department, University College of Northern Denmark, Aalborg, Denmark; University of Florence, ITALY

## Abstract

**Introduction:**

Assessment of balance is key to identifying individuals with postural control deficits and an increased fall risk. Subjects may compensate for their deficits by utilizing other strategies; to avoid this, it is recommended to assess postural control using a dual-task test. In most dual-task tests, it is difficult to monitor the performance in the secondary task and the individual’s task prioritisation. This study evaluated a new dual-task testing approach.

**Materials and methods:**

A convenience sample of 54 community-dwelling elderly (age 65+ years) and a reference group of 20 young participants were included in the study. They performed a test in which they could utilize cues to improve their baseline performance, provided their level of postural control allowed them residual attention capacity for this cognitive task.

**Results:**

Significant performance differences were seen between the young and the elderly. The young group improved their performance time by 23.9% (10.7) and 7.1% (14.2) with a cue and a reverse cue, respectively, whereas the elderly failed to improve their performance time. The test was unable to distinguish between individuals within the elderly group due to a floor effect.

**Discussion:**

The test reveals an individual’s capacity to use cues for anticipatory postural control strategies in a dual-task setting and thereby estimates automatization of postural control. While the young subjects were capable of improving their performance during dual-task conditions, the elderly subjects apparently had no residual attentional capacity allowing them to utilize the facilitating cues. Within the elderly group, the dual-task aspects of the test added no value with respect to differentiation in the level of postural control.

## Introduction

Assessment of balance in the elderly population is a priority for identification of individuals with postural control deficits and increased fall risk [1]. Postural control is often considered to be automatic and to require minimal attention, but there may very well be significant attention requirements, depending on the task and environment and the individual’s age and balance abilities [2]. Many tests used to assess physical performance and balance allow subjects to compensate for their deficits by utilizing other control strategies (e.g., visual and/or cognitive regulation of task performance) [3]. Emphasis has therefore been placed on the importance of assessing both motor and cognitive functioning among elderly at risk of falls by use of dual-task tests [4]. According to a consensus paper (2018) from the Canadian Consortium on Neurodegeneration in Aging: “A new paradigm is emerging in which mobility and cognitive impairments, previously studied, diagnosed, and managed separately in older adults, are in fact regulated by shared brain resources. … This new paradigm requires an integrated approach to measuring both domains” [5].

A dual-task test may comprise monitoring of a standardised primary motor task such as gait speed during walking or sway pattern in a standing position. The secondary task may be a cognitive task or an additional motor task [6]. Good automatization of dynamic postural control during the primary task allows the participant to use any residual attentional capacity for a secondary cognitive task. Most often, the outcome measure is the decrement in the performance in the primary task at concurrent secondary task. The individual’s task prioritisation is difficult to control, however, and the performance in the secondary task should also be monitored. The interpretation of the test is difficult, and it remains challenging to standardise and monitor dual-task testing in a clinical setting [4].

In an attempt to find a new approach to dual-task testing, we developed a test procedure in which a leading (cognitive) cue would allow the participant to improve (motor) performance [7]. This test challenges the participant’s postural control and provokes anticipatory stepping strategies, as the participant must touch different lights placed out of reach. The lights are turned on in a given sequence and may have a colour code (a cue) directing the participant to the placement of the next light. The cognitive task consists in analysing and utilizing the leading cues of the lights for better anticipatory strategies, thereby obtaining a better overall performance time. An improved performance time indicates that postural control in the motor task is automated and that a residual attentional capacity is available for the cognitive task. The assessment approach has been validated with respect to its ability to discriminate between age groups [7]. In the present study, this approach was further developed into a simpler set-up with fewer lights and repetitions.

The aim of the study was to evaluate the new dual-task testing procedure with respect to reliability, criterion validity and construct validity. As the test is intended to measure age-associated balance impairment, the construct validity was evaluated by the ability of the test to discriminate older from younger subjects. Within the group of elderly construct validity was evaluated with respect to fall history and criterion validity was evaluated with reference to the “Timed Up and Go” test and the “Fall Efficacy Scale” questionnaire. Reliability of the test was evaluated by a retest in a subgroup of the elderly.

## Materials and methods

A convenience sample of 54 community-dwelling elderly (age 65+ years) and a reference group of 20 young participants were included in the study. Thirty-six participants from the elderly group participated in a re-test after one week. The elderly participants were recruited from community centres and included if they were older than 65 years and independent with respect to activities of daily living. The exclusion criteria were: dependency on walking aids, significant pain limiting daily functions, known uncorrected visual or vestibular problems, or cognitive impairment (i.e., Mini Mental State Examination (MMSE) < 23) [8]. The young adults were included if they had no known disease or need for medication.

The elderly participants filled out a questionnaire to characterise their fear of falling, the Fall Efficacy Scale (FES), and were asked about their fall history within the past 12 months. FES is a 16-item questionnaire on the concern about falling during indoor and outdoor activities with the score of 64 expressing the highest concern [9]. The participants general physical performance was evaluated by the “Timed Up and Go” test (TUG). TUG is a timed test on physical function and balance in which the participant will raise from a chair, walk three meters, and return to the chair. A performance time of 13.5 seconds has been suggested as a cut-off value with respect to increased fall risk in a community-dwelling population [10].

The Ethics Committee of the North Denmark Region approved the protocol, and the participants provided their written informed consent to participate in the study in accordance with the consent procedure of the regional ethics committee.

### Testing procedure

**Fig 1:**
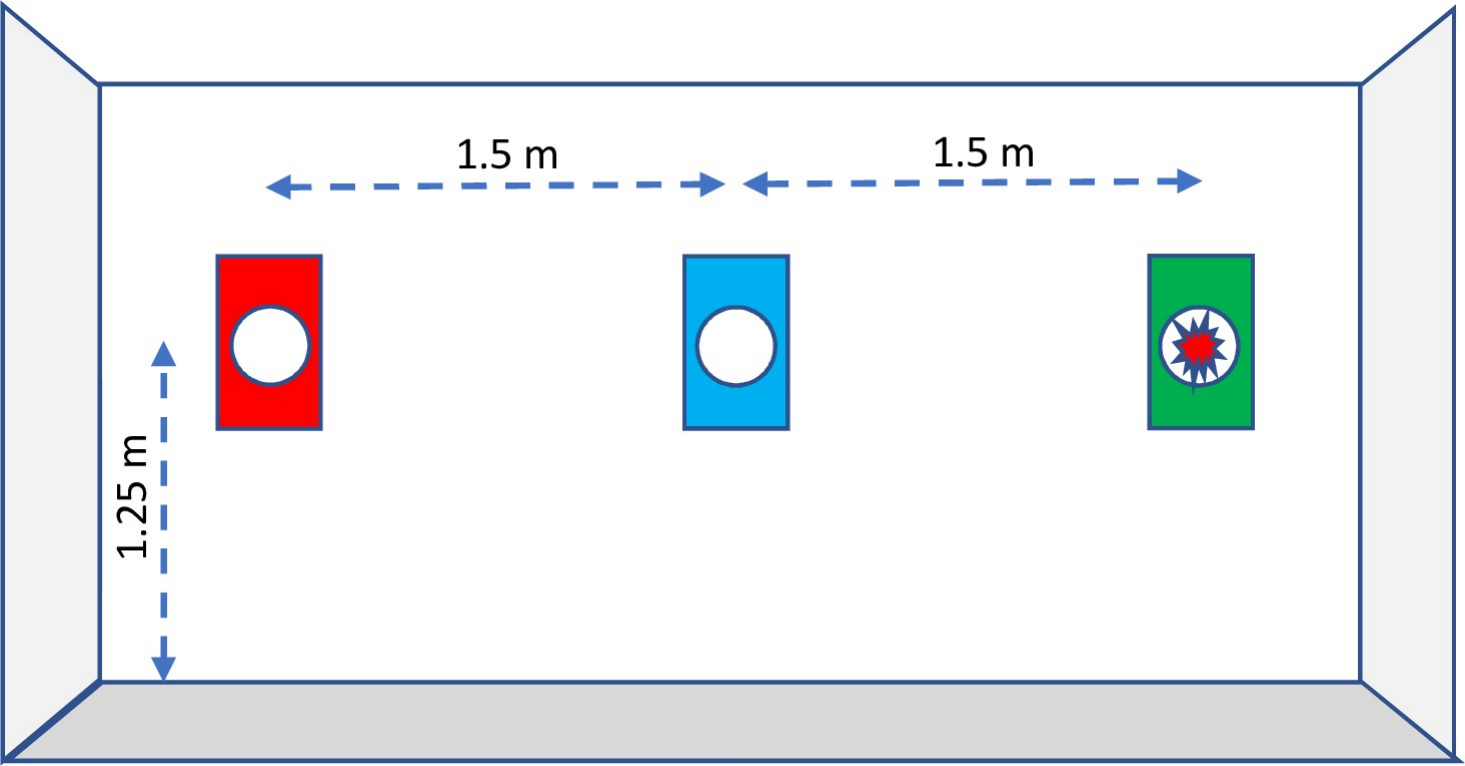
Setup. Lights/sensors are placed on a wall in three zones marked red, blue and green from left to right. They are beyond reach for a person positioned at the centre of the field. When one light appears, it can be switched off by the participant and the next lights will then appear. The light sequence may be either random (single task) or the colour of the light may indicate the zone in which the following light will appear (dual task).

Three lights with inbuilt sensors were placed on the wall at a height of 1.25 m and with a mutual horizontal distance of 1.5 m. Attached to the wall behind each light was a coloured A3 paper which indicated a specific colour zone. From left to right, the colour zones were red, blue and green (see Fig 1). To reach the lights, the participant would need to reach beyond the limit of stability and employ stepping strategies. In this manner, they were challenged in a variety of feedforward postural control strategies in different directions.

The test consisted of four trials with a motor task that remained the same throughout all trials, and a cognitive task that differed from trial to trial in its demand on cognitive resources. In each trial, the motor task consisted of 15 repetitive reaching tasks in which the participants were to turn off the light by touching or holding a hand in front of the light/sensor at a distance of less than 10 cm. The lights were lit one at a time, and once the light of one sensor had been turned off, the subsequent sensor would light up after a 0.5-second delay. The number of lights activated in the different zones was equally divided.

The four test trials were different with respect to cognitive demands. In the first and last trial, the lights were lit in a random order. In trials 2 and 3, the participant was provided with cues that allowed anticipatory motor strategies provided the participant had a residual attentional capacity to utilise these cues. Trial 3 added an extra cognitive load for utilising the cues.

Trial 1. The lights were lit in a random order using one of three colours (red, green and blue). No cue was given as to where the next light would appear. This trial would mainly challenge the participant’s reaction time and postural control.

Trial 2. The colour of the light indicated the position of the next light. If the light was red, the following light would be lit in the red sector. If the light was green, the following light would be lit in the green sector. If the light was blue, the following light would be in the blue sector. With an automated postural control, the participant was expected to possess residual attention capacity allowing him or her to utilise these cues and improve performance time.

Trial 3. The colour of the light indicated the position of the next light, but the red and green cues were reversed. If the light was red, the following light would be in the green sector. If the light was green, the following light would be in the red sector. If the light was blue, the following light would be in the blue sector. Like in trial 3, the cues could be utilised with residual attentional capacity, but the cognitive demands were higher.

Trial 4. Identical to trial 1.

Before each trial, the participants were introduced to the procedure by instruction and demonstration, and they had to turn off five lights to become familiar with the task. They were asked to turn off the lights as fast as possible while still keeping a safe balance. No pauses were planned between the trials apart from time for instructions, and the whole test would last approximately 10 minutes for the elderly participants.

**Fig 2:**
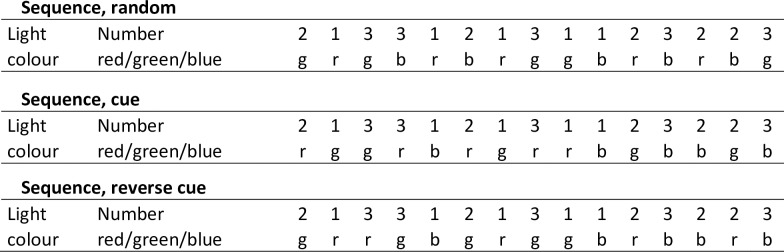
Codes for colours and sequences. Programming the FitLight controller for the three different trial-setup in the test. Colour of the light: red (r), blue (b) and green (g); and sequence number of the light (numbered from left to right).

The lights and the software used for the test were a commercially available product from the FitLight Sports Corp. Ontario, Canada. The FitLight Trainer comes as a wireless system unit comprising eight LED powered lights controlled by a tablet. The system may be programmed for specific sequences of light and colour activation. The lights have an inbuilt sensor that reacts to proximity or touch and deactivates the light. The timing of light deactivation is recorded by the tablet. The programmed sequences of the lights are shown in Fig 2. In each trial, the program was started at a random point of the sequence.

### Data analysis

The performance time for each trial was recorded automatically by the FitLight software and displayed on the tablet-controller of the system. Figures were manually entered into MS Excel, and the statistical analysis was performed in SPSS 25.0.

For each group and session, the averaged performance times of the trials were presented. The mean score of trials 1 and 4 (trials with random light sequences) represented an individual baseline performance. The individual relative percentage changes from this baseline to the trials with a cue and with a reversed cue (trials 2 and 3) were calculated and presented with positive values indicating the relative improvement in performance time (%).

The normal distribution of data was evaluated by QQ-plot and the Kolmogorov Smirnov test. The data from the first random trial and the reverse cue trial in the elderly group was not normally distributed, and non-parametric statistics were therefore used to compare groups and to evaluate correlations between TUG, FES, age and the performance scores.

The inter-day reliability of the four test sequences was evaluated in terms of absolute differences between sessions and by intraclass correlation coefficients (ICCs). ICCs were calculated (after log transformation of the data) by a two-way mixed effects model (ICC 3,1) using absolute agreement. These values were interpreted with the labels assigned by Portney and Watkins [11] where values below 0.75 indicate poor to moderate reliability, values above 0.75 indicate good reliability, and values should exceed 0.90 for many clinical measurements. Furthermore, standard error of measurements (SEM = SD*√(1-ICC)) and minimal detectable change (MDC = 1.96*SEM*√2) were calculated.

## Results

The elderly group had a mean age of 75 (6) years; BMI 27.8 (5.7) male/female distribution 15/39. Twelve persons had fallen within the previous 12 months (22%). The mean FES score was 22.0 (10.3), and the mean TUG was 7.4 seconds (2.8). The young group had a mean age of 23 (1) years and a male/female distribution of 9/11.

### Validity

**Fig 3:**
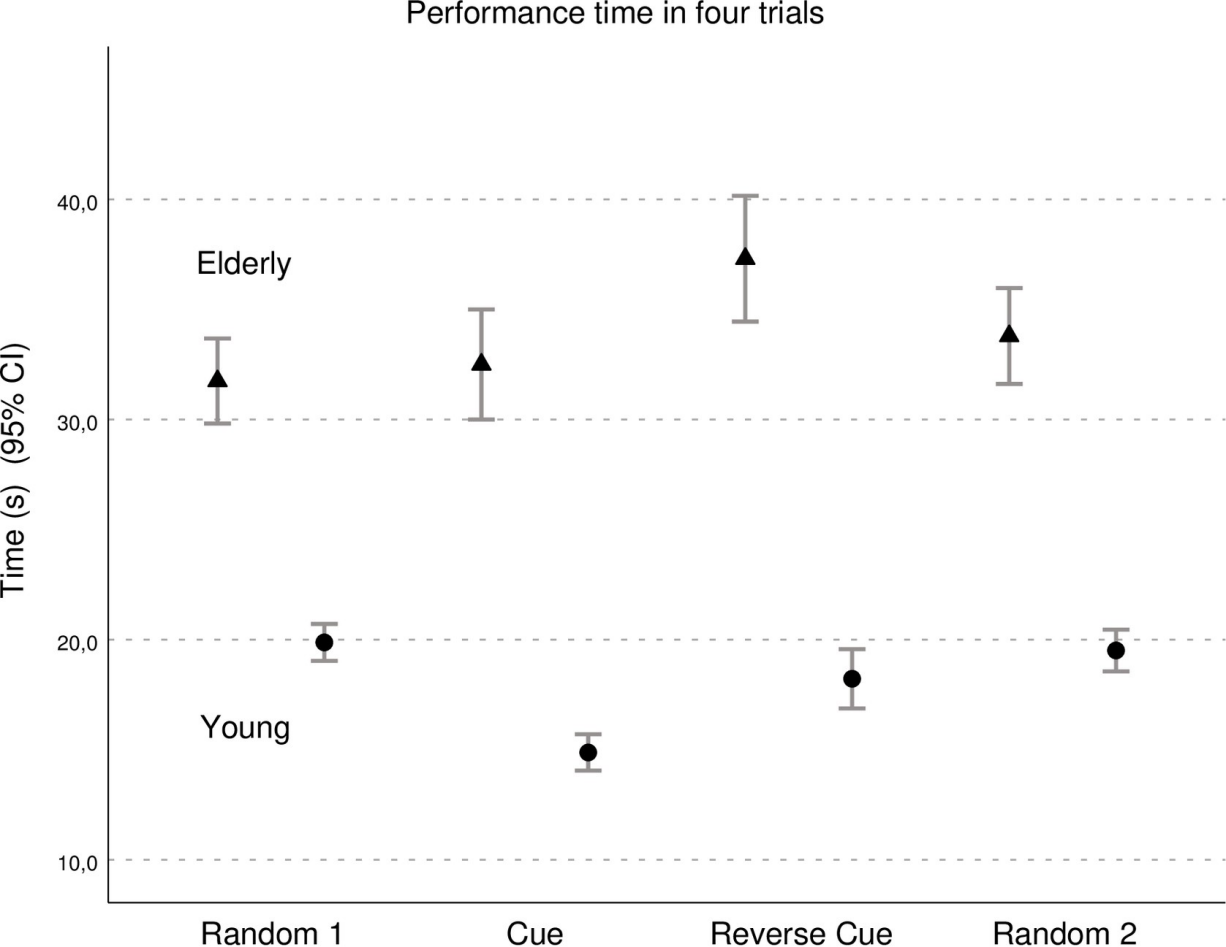
Performance time. The young people performed faster with a cue, while the elderly did not. In tests the elderly were slower than the young.

In general, the elderly group recorded longer performance times during all trials than the young group (Fig 3). Furthermore, only the group of young people improved their performance time when they were given a leading cue or a reversed leading cue. The performance of the elderly did not improve in either of the trials with cues, and it even tended to deteriorate in the trial with the reversed cue (Table 1).

**Table 1:** Performance time(s) and relative improvement with cues (%).

	Random (trial 1 and 2)	Cue	Improvement Cue (%)	Reverse cue	Improvement Reverse cue (%)
Elderly (n = 54)	32.8 (7.3)*	32.5 (9.2)*	0.7 (18.4)*	37.3 (10.5)*	-14.2 (21.3)*
Faller (n = 12)	38.1 (6.7)^¤^	38.5 (9.7)^¤^	-1.8 (21.2)	41.3 (9.9)	-8.3 (17.2)
Non-faller (n = 42)	31.3 (6.8)	30.8 (8.3)	1.4 (17.8)	36.2 (10.5)	-15.9 (22.2)
Young (n = 20)	19.7 (1.8)	14.9 (1.8)^#^	23.9 (10.7)	18.2 (2.9)^#^	7.1 (14.2)

Mean (SD) in seconds and percentage improvement.

*p<0.01, difference between young and elderly.

^¤^p<0.05, difference between fallers and non-fallers.

^#^p<0.05, improvement compared with Random.

The performance time in the four trials correlated, in part, with the TUG test and the FES questionnaire on fall efficacy, but the relative improvement in performance with cues did not (Table 2).

**Table 2:** Correlations between general performance measures and age and the test scores in the elderly group.

	Random (mean)	Cue	Improvement cue (%)	Reverse cue	Improvement reverse cue (%)
TUG	0.87*	0.81*	-0.26	0.66*	0.05
FES	0.60*	0.55*	-0.12	0.41*	-0.05
Age	0.47*	0.44*	-0.25	0.43*	-0.16

*p<0.01

### Reliability

Thirty-six elderly people participated in the test-retest study- The group had a mean age of 76 (6) years; BMI 29.0 (5.1) and male/female distribution 12/24. Nine persons had fallen within the previous 12 months (25%). The mean FES score was 21.1 (7.9), and the mean TUG was 7.8 seconds (2.6). The intra-class correlation coefficients indicated “good reliability”, but a statistically significant improvement in performance time was seen in each of trials after one week (Table 3). The reliability of the relative improvements with cues was not calculated due the floor effect in the test.

**Table 3:** Test-retest scores for the four trials (s) and relative improvements (%) for the elderly (n = 36).

	Test	Retest	ICC	SEM	MDC
Random 1	32.7 (6.6)	30.9 (5.3)*	0.86	0.12	0.33
Cue	34.8 (7.8)	31.9 (7.9)*	0.83	0.14	0.39
Reverse cue	38.8 (9.8)	35.1 (8.0)*	0.78	0.17	0.48
Random 2	35.3 (7.5)	32.5 (7.1)*	0.88	0.11	0.29
Diff Cue	-3.5 (19.1)	-1.6 (23.7)			
Diff Revers cue	-14.6 (19.0)	-11.6 (20.2)			

Mean scores (SD).

*p< 0.05

## Discussion

The young participants improved their performance time in the test when they were provided with a leading cue to facilitate their anticipatory postural control strategies; the elderly did not. The relative changes in performance time for young and elderly people when given a cue and a reverse cue are comparable to previous findings [7]. The simpler test setup used in this study seems to provide the same challenges and yield the same results as the previously published test, which had more lights and more repetitions.

While the young group improved their performance time as expected, when they were given leading cues, the elderly participants did not. The elderly people were apparently unable to utilise this opportunity for more efficient anticipatory strategies. Instead of predicting the next light placement and improve their performance time, they seemed to be confused by these trials. The elderly may have a reduction in attentional control skills and may lack the ability to prioritize the task of combining the light colour with the placement of the following light [12]. The poorer performance may also be ascribed to the concurrent challenge of the dynamic postural control task. The postural control during the motor task of reaching out for the lights may be more difficult and attention demanding for the elderly. This lack of automaticity in the elderlies’ postural control will occupy attention resources and result in a lack of residual attentional capacity for the cognitive task [13].

The elderlies’ performance time in the four trials correlated with the TUG and FES results as well as with the age, as expected. But the relative improvements during tests with cues had no correlation with these measures. This may be ascribed to a “floor-effect” in these elements of the test. The parts of the test designed to reveal the residual attention capacity by including dual-task elements were apparently too difficult for the elderly participants. The test was therefore unable to discriminate between postural control performance levels within the elderly group.

The test discriminated between the groups of elderly and young people. Differences between groups were statistically significant both for the simple motor task with the lights presented in a random order and the parts of the test with dual-task aspects in which the lights provided a leading cue for improvement of the participants’ performance. While it was possible to discriminate between young and elderly people in all four test trials, no benefit was gained from incorporating the dual-task aspects, and the trials with cues provided no additional information about the performance level within this group of elderly people. The dual-task improvements were not statistically different between fallers and non-fallers, and they did not correlate with neither the age nor the TUG and FES scores. The dual-task approach may not be useful for assessing performance level within this population. Similar conclusions were reached in a meta-analysis on dual-task walking paradigms in which the findings indicated that single and dual-task tests of gait speed are equivalent with respect to prediction of falls in older people [14]. Likewise, the ability of the TUG to predict falls in community-dwelling adults is not enhanced by adding a secondary task when performing this test [15].

As a floor effect and high random error appeared in the relative (percentage) changes with cues and reverse cues, these aspects of the test were not evaluated with respect to reliability. The intra class coefficients of the four individual trials of the test indicated good reliability. However, the results also showed significant improvements in the retest performance times after one week. These differences may be ascribed to a learning effect. Apparently, the participants could have been better familiarised with the test before it was used for the assessment of postural control.

## Conclusion

We have developed a test that incorporates motor tasks and cognitive tasks and provides a common performance score measuring anticipatory postural control. The test can discriminate between young and elderly in both single-task and dual-task elements. Whereas the young people were capable of improving their performance during dual-task conditions, the elderly people apparently had no residual attentional capacity to utilise the facilitating cues provided. With respect to differentiation in the level of postural control within the elderly group, the dual-task aspects of the test added no value. In its present form, the test is not useful in a population of elderly people in which the participants are confused by the cues. The test may be used in a population with a higher cognitive ability and more automatized postural control, but a different validation is needed to clarify this.

## Supporting information

S1 Dataset Baseline data and test performance scores for a group of elderly people and a young reference group. Re-test performance scores are provided for a subgroup of the elderly.
(XLSX)
